# Bacteriophage 434 Hex Protein Prevents RecA-Mediated Repressor Autocleavage

**DOI:** 10.3390/v5010111

**Published:** 2013-01-09

**Authors:** Paul Shkilnyj, Michael P. Colon, Gerald B. Koudelka

**Affiliations:** Department of Biological Sciences, University at Buffalo, Buffalo, NY 14260, USA; E-Mails: shkilnyj@gmail.com (P.S.); mpcolon@buffalo.edu (M.P.C.)

**Keywords:** bacteriophage, RecA, lysogeny

## Abstract

In a λ^imm434^ lysogen, two proteins are expressed from the integrated prophage. Both are encoded by the same mRNA whose transcription initiates at the P_RM_ promoter. One protein is the 434 repressor, needed for the establishment and maintenance of lysogeny. The other is Hex which is translated from an open reading frame that apparently partially overlaps the 434 repressor coding region. In the wild type host, disruption of the gene encoding Hex destabilizes λ^imm434^ lysogens. However, the *hex* mutation has no effect on lysogen stability in a *recA^−^* host. These observations suggest that Hex functions by modulating the ability of RecA to stimulate 434 repressor autocleavage. We tested this hypothesis by identifying and purifying Hex to determine if this protein inhibited RecA‑stimulated autocleavage of 434 repressor *in vitro*. Our results show that *in vitro *a fragment of Hex prevents RecA-stimulated autocleavage of 434 repressor, as well as the repressors of the closely related phage P22. Surprisingly, Hex does not prevent RecA‑stimulated autocleavage of phage lambda repressor, nor the *E. coli* LexA repressor.

## 1. Introduction

Upon infection of a host cell, the lambdoid phages choose between two developmental fates, opting either to replicate and lyse the cell, or to enter the latent, or lysogenic phase in which the phages’ chromosome is integrated into that of the host and is replicated along with it [1,2]. Maintenance of the lysogenic state requires the activity of the phage’s cI repressor protein. The repressor functions to maintain lysogeny by repressing transcription from promoters P_R_ and P_L,_ thereby preventing synthesis of proteins needed for phage lytic development. At the same time, the repressor activates transcription of its own gene by stimulating transcription from P_RM_ and thereby maintains appropriate repressor concentrations inside the cell [1].

The survival of the integrated prophage is linked to the fitness and survival of its host. Thus to ensure their survival, the lambdoid phages employ mechanisms that allow prophages to enter the lytic growth pathway and thus escape from hosts whose survival is in doubt. The best understood regulator of lambdoid prophage induction is the RecA protein [3,4,5]. As the master regulator of the SOS response, RecA inactivates LexA protein, the repressor of the SOS response genes, by stimulating its nascent autocleavage activity [5] thereby eliminating LexA protein’s DNA binding activity. Similarly, RecA also stimulates autoproteolysis of the lambdoid phage repressors [6,7]. When the repressor is cleaved into its constituent carboxyl terminal and amino terminal domains, it is incapable of binding DNA with high affinity DNA binding or forming higher order oligomers (dimers, tetramers, *etc.*) Consequently transcription from P_R_ and P_L_ are no longer repressed, and the prophage enters the lytic growth pathway.

Transcription from P_RM_ requires the stimulatory activity of DNA bound repressor. Hence this promoter is only active in the presence of repressor, *i.e.*, in the lysogenic state. In addition to the repressor gene many lambdoid bacteriophages encode additional proteins on the transcript initiated from P_RM_. Since these genes are not essential for lysogenic or lytic growth, they are considered accessory [8]. However in some cases they apparently provide an advantage to both the host and the phage. For example, the RexA and RexB proteins, which are produced by bacteriophage λ lysogens, excludes superinfection by bacteriophage T4rII^−^ [9]. In bacteriophage 933W a eukaryotic-like tyrosine kinase is cotranscribed with cI repressor [10] and its activity aborts infection of the 933W lysogen by a superinfecting HK97 phage [11]. Further examples of proteins that are expressed during lysogeny of temperate bacteriophages are found in the tripartite immunity system of phages P1 and P7 [12], and phage K139 [13]. These proteins also prevent superinfection by other phages.

In a lysogen, bacteriophage 434 expresses, in addition to cI repressor, a second protein Hex, which is encoded on the mRNA transcript initiated from P_RM_. Susskind and Botstein demonstrated that *S. typhimurium* lysogenized with λ^imm434^ excluded growth of superinfecting phage P22 [14]. However, *S. Typhimurium *lysogenized with λ^imm434:Hex^^−^ did not exclude the growth of superinfecting phage P22. The name Hex, therefore, stands for heterologous exclusion.

Since the heterologous phage exclusion functions of each of the proteins encoded along with repressor is apparently highly specific for particular phage(s), we wondered whether these proteins may have a more general role in bacteriophage biology. Our results show that deletion of the open reading frame of Hex increases the spontaneous induction frequency of λ^imm434^ phage. We also show that in a RecA^−^ strain of *E. coli*, the spontaneous induction of λ^imm434:hex^^−^ and wild type λ^imm434^ are identically low. The results of genetic and biochemical experiments suggest that, when present, Hex increases the stability of bacteriophage 434 by interfering with RecA-mediated autocleavage of the phage 434 repressor. Consistent with this finding, we found that purified Hex blocks RecA-mediated stimulation of 434 repressor autocleavage. We found that Hex also prevents RecA stimulated autoproteolysis of the repressor encoded by bacteriophage P22, but not the repressor of bacteriophage λ or *E. coli *LexA. All of which are susceptible to RecA stimulated autoproteolysis. This finding suggests that Hex does not block RecA activity by directly interacting with it.

## 2. Results

**Figure 1:**
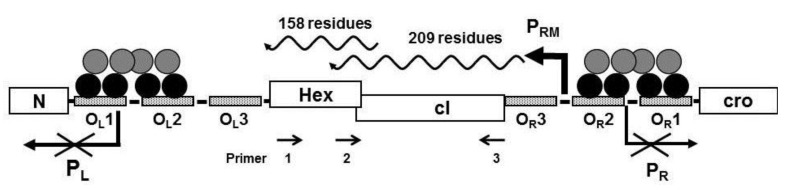
Schematic representation of the immunity region of bacteriophage 434. The N- and C-terminal domains of the cI repressor dimers are represented by black and gray circles, respectively bound to the operator sites. In above configuration shown, transcription of the promoters P_R_ and P_L _are repressed and transcription from P_RM_ is activated on. The Hex gene is located on the same transcript initiated at P_RM_. Primers designated 1, 2, and 3 were used to analyze the P_RM_ transcript (see text).

**Figure 2:**
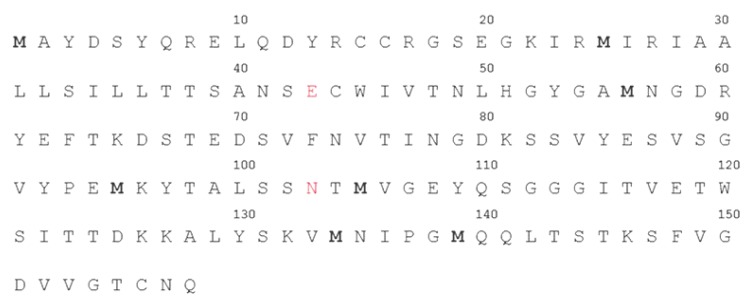
The putative amino acid sequence of Hex. The predicted amino acid sequence starts from the 5'-most AUG, in the hex transcript. Additional potential methionine initiators highlighted. The red amino acids highlight the beginning and end of the sHex polypeptide (see also Figure 5).

**Figure 3:**
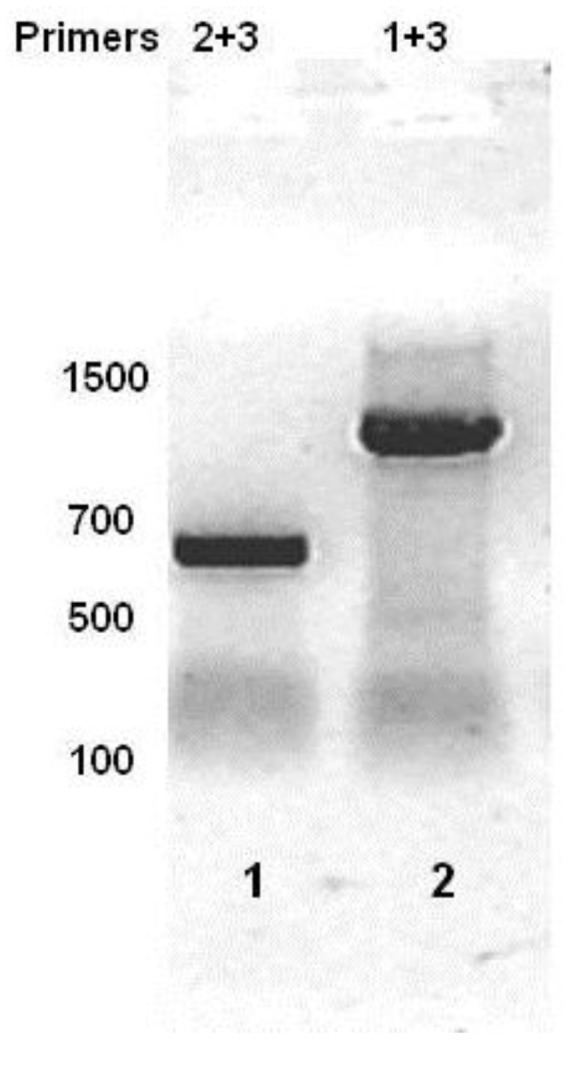
RT-PCR analysis of total bacterial RNA isolated from MG1655::λ^imm434^ lysogens. Total RNA was reverse transcribed using Primer 1 (see Figure 1). The cDNA was PCR separately amplified with primers 2 + 3 (lane 1) or 1 + 3 (lane 2), and products separated on an agarose gel. The numbers represent sizes of molecular weight standards.

Computer analysis of the three reading frames in the mRNA initiated from P_RM_ of the bacteriophage 434 identified a a potential open reading frame with a capacity to code for a 158 amino acid protein located 3’ to the 434 repressor gene (Figure 1). The predicted amino acid sequence of the identified ORF is shown in Figure 2, beginning with a methionine encoded by the 5'-most AUG of the transcript. To ensure that the transcript initiated at P_RM_ extends through the putative ORF downstream from the repressor gene, we isolated total bacterial RNA from MG1655(λ^imm434^) lysogens, reverse transcribed this RNA using primer 1 (Figure 1) and the resulting cDNA was analyzed by PCR using a series of primers complementary to this region (Figure 1). Since the RNA was isolated from a phage lysogen, RNA encoding the repressor must be present. As expected, a product of ~627 bp, the predicted size, was obtained when primer set complementary to this region (Primers 2 + 3) of the RNA was used (Figure 3, lane 1). When PCR was performed using primers 1 + 3, a DNA product of about 1100 base pairs was obtained (Figure 3, lane 2). This observation shows that in the MG1655::λ^imm434^ lysogen, transcripts initiated at P_RM_ continue through the predicted Hex ORF.

### 2.1. Effect of Hex Mutation on λ^imm434^ Prophage Induction

**Figure 4:**
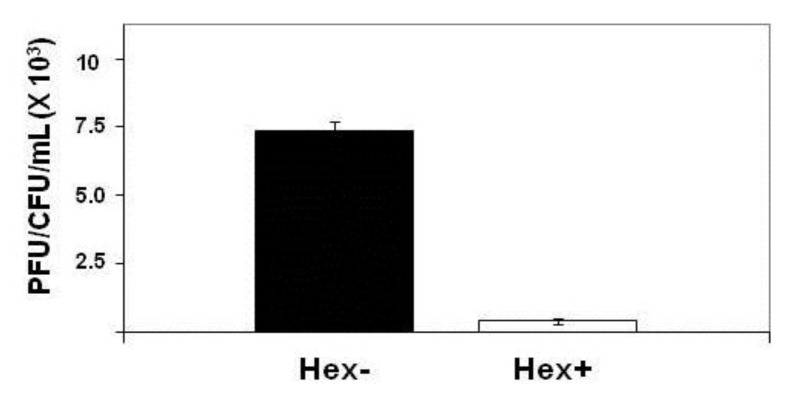
Effect of *hex* mutation on stability of MG1655::λ^imm434^ lysogens. MG1655(λ^imm434^) and MG1655(λ^imm434hex^^−^) were grown to saturation, washed and resuspended in fresh media for 4 hours. The amount of phage release into the culture was determined by plating various amounts of supernatant on MG1655 cells that did not contain λ^imm434^ prophage. The concentration of lysogen cells was determined by plating various dilutions of cells on LB agar plates. Error bars are standard deviation of twenty four separate repeats.

*E. coli* strain MG1655 was lysogenized with λ^imm434hex^^−^ and the amount of phage produced spontaneously by *E. coli* strain MG1655 separated lysogenized with λ^imm434^ and λ^imm434hex^^−^ was determined as described in the Experimental Section. We find that the MG1655::λ^imm434hex^^−^ lysogens produced 30-fold more phage during a five hour incubation then did the wild type (MG1655::λ^imm434)^ lysogens (Figure 4). This finding suggests that Hex may function in regulating the lysis-lysogeny of integrated λ^imm434^ prophage. Alternatively, the increased in number of phage produced during the incubation may be a result of an effect of *hex* on the number of phage released/cell. We tested this idea by measuring phage burst sizes for both of these bacteriophages (see Experimental Section). We find that the burst sizes of the λ^imm434^ and λ^imm434hex^^−^ phages are indistinguishable from each other; under the conditions of our measurements, each cell infected by these phages produces 104 phage. 

### 2.2. Role of RecA and Hex in Regulating Prophage Stability

Since RecA affects the level of spontaneous phage induction in a RecA^+^ host [15], we tested the idea that Hex may affect spontaneous induction by influencing RecA’s co-protease anti-repressor activity. To test this idea, we compared the spontaneous induction frequency of λ^imm434^ and λ^imm434hex^^−^ prophages in wild type and RecA^−^ MG1655 strains. The spontaneous induction frequency of MG1655recA938::cat (λ^imm434hex^^−^) is 1.8 ± 0.08 × 10^−5^, which is nearly identical to the spontaneous induction rate of MG1655recA938::cat::(λ^imm434^) (Table 1). In contrast, the spontaneous induction frequency of λ^imm434hex^^−^ and λ^imm434^ in wild type MG1655 are remarkably different. In the wild type host, the amount of induction of MG1655(λ^imm434hex^^−^) is nearly 30-fold higher than the induction frequency of MG1655(λ^imm434^) (Table 1) Although the frequency of spontaneous induction in a RecA^−^ host is much lower than in the wild type MG1655 host, the nearly identical frequency of induction in RecA^−^ host of both λ^imm434hex^^−^ and wild type λ^imm434^ suggests that Hex increases the stability of λ^imm434^ by interfering with RecA mediated autocleavage of the cI repressor. 

**Table 1:** Effect of *hex* Deletion on Spontaneous Induction of RecA^−^ MG1655(λ^imm434^) Lysogens. The values (+/− standard deviation) were determined as described in the Experimental Section. Data is an average based on twenty four separate repeats.

Strain		PFU/mL/viable cell (X1000)			
Wild-type		0.275 ± 0.06			
*rec*A-		1.8 ± 0.08 x 10^-5^			
*hex-*		7.34 ± 0.1			
*rec*A-/*hex*-		1.8 ± 0.23 x 10^-5^			

### 2.3. Expression and Purification of Hex

**Figure 5:**
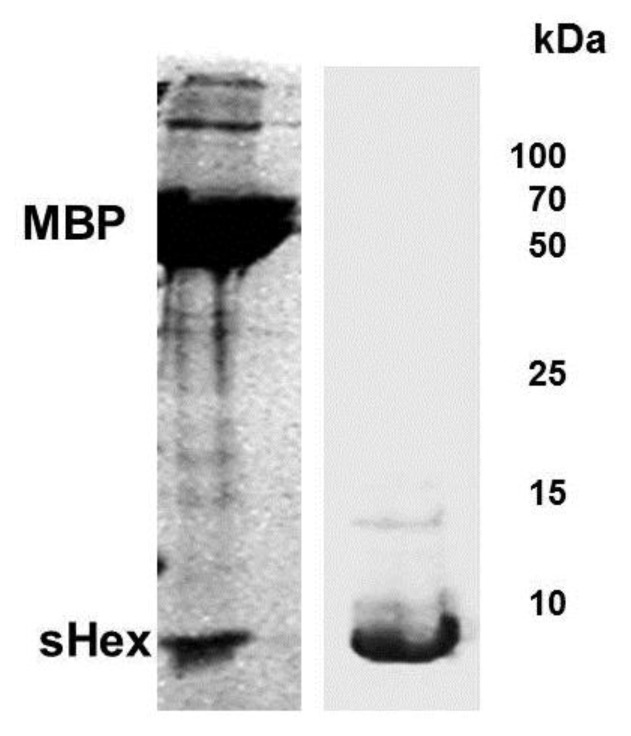
Expression and purification of Hex polypeptide. Full length *hex* gene sequence was inserted into plasmid pMAL-c2X to generate a fusion protein between the genes encoding MBP and Hex. *E. coli* extracts containing the fusion peptide were passed through an amylase column and the eluate digested by Factor Xa (see Experimental Section). Shown is a Coomassie stain of the products of the Factor Xa digest of the eluate from amylase column, fractionated by SDS-PAGE (left panel). Bacterial strain BL21(DE3)[pLysS] was transformed with plasmid psHex containing the gene coding for the identified sHex (see Figure 2). The sHex was purified from cell extracts using ammonium sulfate, GuHCl, followed by size exclusion chromatography. The right panel shows Coomassie stain of an SDS-PAGE of the the pooled protein-containing fractions from the sizing column. The positions of sHex, maltose binding protein (MBP) and molecular weight standards are indicated.

We placed the *hex *gene under control of the T7 RNA polymerase promoter and attempted to express this full length gene product. We repeatedly observed that upon induction of Hex expression, cultures bearing plasmids encoding full-length Hex stopped growing, and subsequently showed a decrease in optical density at 600 nm. Therefore we initially attempted to purify Hex as a fusion with maltose binding protein (MBP). We reasoned that as a fusion with MBP (Maltose Binding Protein), the Hex peptide may be less detrimental to cell viability. When a plasmid encoding a MBP-Hex fusion protein was transformed into *E. coli* strain BL21(DE3)[pLysS], over-expression of the MBP-Hex protein was induced upon addition of IPTG. The expressed peptide was purified on an amylose resin and fractions containing the MPB-Hex were pooled and digested with factor Xa to separate the two proteins and then fractioned on SDS-PAGE (Figure 5). Sequence analysis shows that the MBP-Hex fusion protein contains a single Factor Xa target sequence, located at the fusion site between MBP and Hex. Thus we would have anticipated this treatment would have released a ~17.5 kDa protein-the size of full-length Hex. Instead, treatment of the MBP-Hex protein with Factor Xa produced two fragments, one the approximate size of MBP, and the other roughly 6 kDa (Figure 5). Repeated attempts to generate the full Hex (MW ~16 kDa) from the MBP-Hex fusion by cleaving the fused peptide with the Factor Xa under various conditions, or sources of Factor Xa failed. We are unsure as to why we are unable to obtain full length Hex by Factor Xa treatment. However we hypothesized that this 6 kDa protein fragment represented a stable fragment of Hex and proceeded to analyze this Hex fragment. 

### 2.4. Characterization of sHex

To determine whether the 6 kDa protein fragment that results from factor Xa cleavage of the MBP‑Hex fusion is a fragment of Hex, we cut this fragment out of a 15% SDS-polyacrylamide Tris‑Tricine gel, and determined its sequence by MALDI-TOF mass spectrometry. The resulting sequence confirmed this peptide derives from full length Hex. The shortened Hex polypeptide (hereafter referred to as sHex) sequence begins with glutamic acid 43 and ends with asparagine 103 (see Figure 1). 

**Figure 6:**
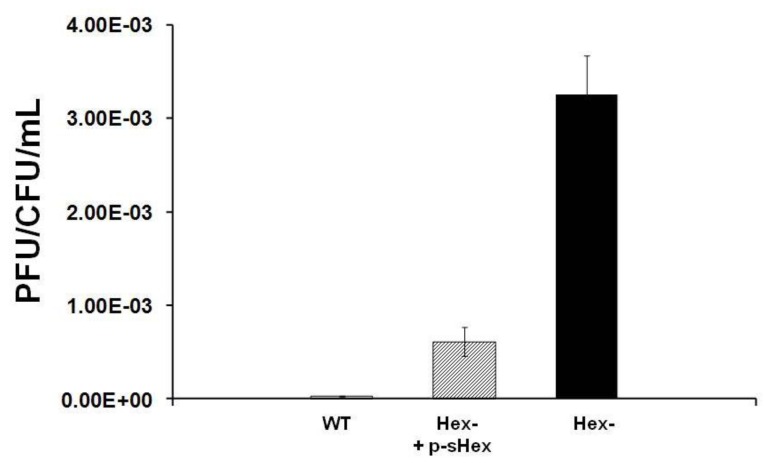
Effect of sHex expression on the stability of MG1655::λ^imm434 hex^^−^ lysogens in MG1655. MG1655::λ^imm434 hex^^−^ lysogens were transformed with a control plasmid (black bars) or a plasmid encoding the sHex only (hatched bar). For comparison, the amount of spontaneously released from MG1655(λ^imm434^) lysogens (denoted as WT) under these conditions is also shown (white bar). The amount of phage spontaneously released was determined as described in the Experimental Section. Error bars are standard deviation of twenty four separate experiments.

We determined whether sHex is capable of complementing the effects of Hex deletion. To accomplish this, we amplified DNA encoding the sHex sequence and placed expression of sHex gene under the control of the T7 RNA polymerase promoter in pET17b [16], creating the plasmid p-sHex. The sequence of this protein is as given in Figure 2, except it contains an initiator methonine precedingE43. MG1655(λ^imm434hex^^−^) lysogens were transformed with p-sHex together with pGP1-2 which encodes a T7 RNA polymerase under the control of the temperature sensitive *cI857* mutant λ repressor [16]. As a control, MG1655(λ^imm434hex^^−^) cells were transformed with pGP1-2, and pET17b lacking the sHex insert. Cultures of these cells were grown to saturation overnight at 32 °C, washed, and resuspended in fresh medium and incubated at 37 °C for four hours. After 4 hours, the spontaneous induction frequencies of λ^imm434hex^^−^ prophages in MG1655 with or without the sHex encoding plasmid were determined as described (Experimental Section). Expression of sHex reduces the spontaneous induction frequency of Hex^- ^lysogens by ~7-fold compared to cells transformed with control plasmid (Figure 6). This finding indicates that sHex can act in *trans* to partially complement the *hex- *defect in λ^imm434hex^^−^. This observation suggests that sHex constitutes the ‘active’ fragment of Hex.

### 2.5. sHex Inhibits RecA-Mediated Autocleavage of 434 Repressor *in Vitro*

**Figure 7:**
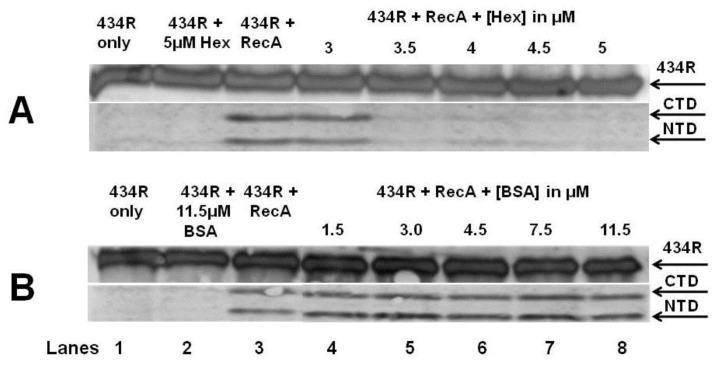
Effect of Hex on RecA-stimulated autocleavage of 434 Repressor. (**A**) 434 repressor [300 nM] (lane1) was incubated with sHex (lane2), 8 µM RecA (lanes 3) or 8 µM RecA plus the indicated sHex concentrations (lanes 5–8). (**B**) In identical procedure as in (A), except BSA was substituted for sHex. The figure is a composite immunoblot of the reaction products reacted with anti-434R antibodies and visualized by chemiluminescence.

The results in Table 1 suggest that Hex may alter the spontaneous induction frequency of λ^imm434^ prophages by interfering with RecA mediated autocleavage of the 434 repressor, thereby reducing the amount bacteriophage 434 produced. To test this hypothesis we purified sHex as described in the Experimental Section and examined the effect of this protein on RecA-mediated autocleavage of 434 repressor. Mixing sHex (5 µM) with 300 nM 434 repressor does not result in the formation of any 434 repressor cleavage products (Figure 7A, lane 2), whereas mixing 434 repressor with RecA (see Experimental Section) causes the formation of two lower molecular weight antibody reactive species (Figure 7A, lane 3). The molecular weights of these products correspond to the 434 repressor N- and C-terminal domain fragments, consistent with the previous observation that RecA stimulates 434 repressor autocleavage [17]. When increasing concentrations of sHex are added into mixtures of 434 repressor and RecA, the amount of repressor cleavage products observed progressively decreases (Figure 7A, lanes 5–8). In an identical set of experiments in which increasing concentrations of BSA instead of sHex were added into the mixtures of 434 repressor and RecA, BSA did not interfere with the reaction (Figure 7B) Therefore the findings in Figure 7 indicate that sHex interferes with the ability of RecA to catalyze autoproteolysis of 434 repressor *in vitro*. 

### 2.6. Effect of sHex on RecA-Mediated Autocleavage of Related Repressors P22, λ, and the Bacterial LexA Repressor

**Figure 8:**
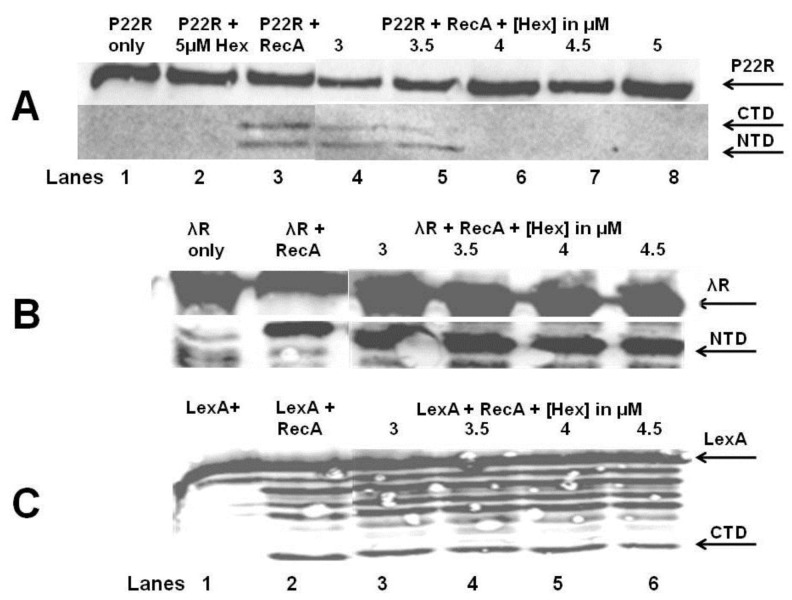
Effect of Hex on RecA-stimulated autocleavage of bacteriophage and bacterial repressors. Bacteriophage repressors from phage P22 (**A**), λ (**B**), and the bacterial SOS repressor LexA (**C**) were incubated in the absence (lane1) or presence of indicated concentrations of sHex and/or 5 µM RecA. The figure is a composite immunoblot of the reaction products reacted with anti-protein antibodies and visualized by chemiluminescence.

To determine whether or not the sHex inhibition of RecA stimulated autoproteolysis is specific to the 434 cI repressor, we examined the effect of sHex on RecA stimulated autocleavage of related cI repressors from lambdoid phages P22 and λ, as well as the SOS repressor LexA, which also undergoes RecA stimulated autocleavage. In an identical procedure as described above, we examined the effect of adding sHex on RecA stimulated autocleavage of P22 repressor (Figure 8A), λ repressor (Figure 8B) and LexA repressor (Figure 8C). Similar to the case with 434 repressor, added sHex interferes with the RecA stimulated autocleavage of P22 repressor (compared Figure 8A with Figure 7A). In contrast, added sHex does not inhibit RecA mediated autocleavage of the λ repressor (Figure 8B) under these conditions. Similar to the λ repressor case, sHex is also incapable of blocking RecA stimulated autocleavage of the SOS repressor LexA (Figure 8C) under these conditions. 

## 3. Discussion

Our results show that the Hex protein of bacteriophage 434 reduces spontaneous induction of the 434 prophage. Based on the results of *in vitro *biochemical data, Hex apparently reduces prophage spontaneous induction by inhibiting RecA mediated autoproteolysis of the cI repressor. The *hex* gene is cotranscribed along with that of the cI repressor gene on mRNA that is initiated at the promoter for repressor maintenance (P_RM_) in a *P_RM_-cI-Hex* operon (Figure 1 and Figure 3). Many temperate prophages produce accessory proteins that benefit the phage, the *E. coli* host, or both, from the same message as their repressor. The most common advantage conferred by many accessory proteins is exclusion of other superinfecting phages [9,12,13,18]. Our data show that the Hex protein of bacteriophage 434, in addition to conferring immunity to phage P22 when expressed in *S. typhimurium*, also reduces induction of λ^imm434^ by interfering with RecA stimulated autocleavage of phage 434 repressor. When the RecA protein is not present in *E. coli* host, λ^imm434Hex^^−^ induces with same frequency as the wild type phage (Table 1), however in a wild type *E. coli* host, λ^imm434Hex^^−^ induction is 30 fold higher. Producing sHex from a plasmid in λ^imm434hex^^−^ lysogens reduces the spontaneous induction about 7 fold (Figure 6). These results suggest that Hex is an advantageous adaptation to bacteriophage 434 in that it “fine tunes” the phage lysis-lysogeny decision. 

The host SOS response to DNA damage causes the lysogenic phage to enter lytic growth; once this cycle initiated, it is irreversible. Under normal conditions, (aerated rich media and lack of DNA damaging agents) SOS genes are expressed at a very low basal level [12]. Furthermore, under normal conditions there is enough repressor made to reduce the transcription of P_R_ roughly 1,000 fold [1]. However, since phage 434 cI repressor’s cooperatively binds DNA, a 5 fold drop in repressor concentration causes repressor to only partially occupy O_R_1 and O_R_1, and thus increase the activity of P_R_ by 50% [19]. The small decrease in repressor concentration, combined with a small amount of active RecA, which may lead to further autocleavage of the repressor. This situation could result in unwanted spontaneous induction. Bacteriophage 434 may use Hex to prevent this “false alarm” induction.

The *in vitro *autocleavage experiments shown in Figure 7 and Figure 8 demonstrate that sHex blocks RecA mediated autocleavage of the phage 434 repressor and P22 repressor, but not λ repressor or *E. coli* LexA repressor (a protein central in the SOS response). Although the 434 repressor, P22 repressor, λ repressor and LexA are structurally similar, they exhibit differences in their mechanisms of RecA‑mediated autocleavage. For example, 434 repressor undergoes autocleavage most efficiently as a DNA-bound dimer [17], as opposed to lambda and LexA repressors which can be efficiently autocleaved as monomers [20,21]. P22 repressor autocleavage increases with increase repressor concentrations, which suggests that P22 repressor is a better substrate for RecA-catalyzed inactivation as a dimer [4]. The different mechanisms of intra or intermolecular cleavage are also prevalent in other RecA targets such as UmuD [22]. These differences in RecA mediated autocleavage of various repressors may account for the selectivity of Hex for the repressors of phages 434 and P22. 

RecA function can be inhibited by competing for DNA binding by DNA binding proteins, filament formation, or preventing RecA from hydrolyzing ATP [23]. For example, RecA dependent proteolysis of P22 repressor is blocked when P22 antirepressor-repressor complex is formed, which prevents access of RecA to the P22 repressor [24]. Phage P22 *virA* produces the antirepressor protein [24]. The specific manner in which Hex prevents RecA mediated autocleavage of 434 and P22 repressors suggests that Hex does not function by directly competing with RecA for DNA, does not prevent ATP hydrolysis by RecA, nor does it directly interact with RecA active site for repressor interaction. Rather, Hex may interact with 434 repressor, and its close homolog the P22 repressor (and with repressor‑DNA complexes), thereby preventing RecA mediated autocleavage. Regardless of the precise mechanism, it is clear that Hex reduces RecA-mediated repressor autocleavage. We further speculate that by being selective only to phages 434 & P22 repressors, Hex does not interfere with bacterial SOS repair. A number of DNA repair cycles may be necessary throughout the bacterial life cycles in order to repair minor DNA damage. These repairs will require a small amount of SOS gene products, active RecA being one of them. However the phage must be able to distinguish life threatening DNA damage from more minor effects, and Hex may function in this capacity.

Outside of the biological function of Hex, for puzzling, and as of yet unidentified reasons, overexpression of full-length Hex is lethal to cells. We were also unable to isolate the MBP-Hex fusion. Treatment of the fusion protein with factor Xa, surprisingly, yielded a much shorter peptide then the full length Hex. Neither long nor short Hex contain factor Xa cleavage sites, nor are there any basic residues present at the ends of the sHex which sometimes can be cleaved by factor Xa. However a presence of cryptic Xa sites cannot be excluded. The possibility that full length Hex is processed to sHex by the activity of residual bacterial proteases unrelated to factor Xa present during the purification process also cannot be excluded. A more intriguing possibility is that full length Hex has an intrinsic autoprotease activity, and undergoes spontaneous proteolytic cleavage. Regardless of the precise mechanism of the full length Hex modification, we conclude that we have identified the active Hex peptide, and demonstrated its role in bacteriophage 434 lysis-lysogeny modulation. 

## 4. Experimental Section

### 4.1. Media and Growth Conditions

Liquid cell cultures were grown in Luria broth or M9 minimal media [25], each of which were supplemented with 100 μg/mL ampicillin and 30 μg/mL kanamycin, where needed, to select for the presence of plasmids.

### 4.2. Bacterial Strains, Phages, and Plasmids

The host strain used for all spontaneous induction analysis was MG1655 [26,27]. The MG1655 *recA* mutants were created by P1 transduction using a lysate from *E. coli* GW4212, which bears the *recA938::cat* allele [28] (a gift from Mark Sutton, University at Buffalo, Buffalo, NY, USA). Both wild type and the *recA* mutant MG1655 were lysogenized with λ^imm434^ or λ^imm434: hex^^−^ as previously described [8]. The *hex* mutant version of λ^imm434^ was a gift from Lynn Thomason, Center for Cancer Research, National Cancer Institute at Frederick, Frederick, MD, USA. This phage was generated completely replacing the Hex coding sequence with that encoding the gene for chloramphenicol acyltransferase flanked by its promoter and transcription terminator. The replacement was done by recombineering.

The Hex DNA sequence ATGGCCTATGACTCCTATCAACGGGAACTGCAAGATTATCGGTGTTGTCGTGGAAGCGAGGGTAAAATTCGTATGATCAGGATTGCGGCGCTACTCTCAATACTCTTAACTACCAGCGCCAATTCTGAATGCTGGATTGTCACAAACCTGCACGGGTACGGGGCAATGAATGGCGATCGTTACGAGTTTACAAAAGACAGCACGGAAGATTCCGTTTTCAACGTAACAATAAATGGCGATAAATCATCAGTTTATGAATCAGTTTCTGGCGTCTATCCAGAGATGAAATACACTGCTTTGTCATCGAACACTATGGTAGGAGAATACCAGTCTGGAGGAGGCATAACCGTTGAAACTTGGTCAATCACTACAGACAAAAAAGCTCTTTACTCCAAAGTAATGAACATCCCAGGTATGCAACAACTTACATCAACCAAATCCTTTGTTGGTGATGTAGTCGGAACCTGCAATCAGTAAT was PCR amplified and cloned into XbaI and HindIII sites of pMAL-c2X (New England Biolabs), generating a MBP-Hex fusion. This construct encodes a Factor Xa cleavage site in the linker between Hex and MBP. Expression of this fusion protein is under control of the IPTG (isopropyl-β-_D_-thiogalactopyranoside) inducible *lac* promoter. A truncated version of Hex (short hex—sHex) was created by PCR amplifying the following sequence from the central portion of the hex gene: GAATGCTGGATTGTCACAAACCTGCACGGGTACGGGGCAATGAATGGCGATCGTTACGAGTTTACAAAAGACAGCACGGAAGATTCCGTTTTCAACGTAACAATAAATGGCGATAAATCATCAGTTTATGAATCAGTTTCTGGCGTCTATCCAGAGATGAAATACACTGCTTTGTCATCGAAC using forward primer 5'-GGCCATCATATGGAATGCTGGATTGTC-3', and a reverse primer 5'-CCAGCGCTCGAGTTAGTTCGATGACAAAGC-3' (IDT Technologies). This DNA fragment was cleaved with NdeI and XhoI (New England Biolabs), purified and ligated into pET17b (Novagen) which was cleaved with the same enzymes.

### 4.3. Analysis of RNA Originating from P_RM_

RNA was isolated from MG1655^λimm434^ as described in reference [29]. Briefly, cell cultures were grown to OD_600_ = 0.6, collected by centrifugation at 12,000 × *g* at 4 °C, and resuspended in protoplasting buffer (15 mM Tris-HCl pH 8.0, 0.45 M Sucrose, 8 mM EDTA) containing 50 mg/mL lysozyme, and incubated 15 minutes on ice. Protoplasts were collected by centrifugation for 5min at 5,900 × *g* 4 °C, resuspended in 0.5 mL gram-negative lysing buffer containing DEPC, and incubated for 5 min at 37 °C. The RNA was precipitated from the preparation by adding saturated NaCl (34g/100 mL of water) and 100% ethanol, in an overnight incubation at −20 °C. The RNA was collected by centrifugation and washed with ice-cold 70% ethanol, dried, and dissolved in DEPC‑treated water. The RNA was digested for 10 minutes with RNAse-free DNase I (Promega Madison, WI, USA) which was subsequently deactivated at 65 °C for 10 minutes. The RNA was immediately used in RT-PCR reactions. Synthesis of first-strand cDNA was performed using AffinityScript^TM^ QPCR cDNA Synthesis Kit (Qiagen Valencia, CA, USA). The primer 5'‑CGAGATTGAGGTGGGGATTAC-3' was incubated with 3 μg total RNA, first strand master mix, AffinityScript RT/RNase block enzyme mixture, and incubated at 25 °C for 5 minutes, followed by 15 minutes in 42 °C. The reaction was terminated by placing the mixture at 95 °C for 5 minutes, chilled on ice, and immediately used in PCR with the following primer combinations: 5'-CGCGTCCAGTATCTACTAACA-3' with 5'-CAGCTTGGACTTAACCAGGCT-3', or 5'-CGAGATTGAGGTGGGGATTAC-3' with 5'-CAGCTTGGACTTAACCAGGCT-3'. The PCR products were fractionated by electrophoresis on agarose gels. 

### 4.4. Analysis of Spontaneous Induction

Measurements of spontaneous induction frequency were performed as described previously [30]. Briefly, cultures of MG1655 lysogenized with λ^imm434^ or λ^imm434 hex^^−^ were grown to saturation overnight in LB or M9 minimal media at 37 °C. Phage that spontaneously produced overnight were removed from the stationary cells by washing three times with fresh medium. During the last wash the cells were resuspended in a volume of fresh medium that was equal to the starting culture volume. The cultures were incubated with shaking at 37 °C for 5 hours. Cell cultures were then centrifuged at 8,000 × g, and the phage-containing supernatant was sterilized by addition of chloroform. To determine the number of CFU (colony forming units), an aliquot of the remaining phage culture was evenly spread over Luria broth (LB) solidified with Luria agar (LA) in a Petri dish. The amount of phage released into the supernatant was determined by evenly distributing the released phage on nonlysogenic MG1655 as described previously [8]. The amount of phage released per cell in culture was calculated as the plaque forming units PFU/CFU ratio. 

### 4.5. Measurement of Burst Size

Standard methodologies were used for determining the mean number of phage particles per bacterium [31,32] Briefly, phage particles at a concentration of 1 × 10^7^ pfu mL^−1^ were mixed with 1 × 10^8^ cfu mL^−1^ MG1655 in LB supplemented with 10 mM MgSO_4 _and 5 mM CaCl_2_ for 5 minutes at 37 °C. The mixture was diluted 10,000-fold with pre-warmed LB to give a final culture volume of 10 mL. At intervals up to 75 minutes, two 0.2 mL samples were acquired to determine amount of unadsorbed (U) phage particles and total (T) phage particles. The U phage particles were additionally treated with 2% CHCl_3_. The burst size is calculated as 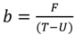 where F is the final phage count at time point 75 minutes and T and U are total and unadsorbed phage, respectively.

### 4.6. Analysis of Complementation of the Hex Mutation

The MG1655 λ^imm434 hex^^−^ strain was transformed with pET17b bearing the short Hex gene under the T7 RNA polymerase promoter, together with plasmid pGP1-2 which contains temperature inducible T7 RNA polymerase [16]. Control MG1655 λ^imm434 hex^^−^ lysogens were transformed with plasmids pGP1-2 and pET17b that does not express any Hex derivatives. The cultures were grown overnight at 32 °C, and the cells collected by centrifugation as described above, washed three times and resuspended in a volume that was equal to the starting culture volume, and incubated at 37 °C for three hours. Cell cultures were then centrifuged at 8,000 × *g* and the phage containing supernatant was sterilized by addition of chloroform. To determine the number of CFU, an aliquot of the remaining phage culture was evenly spread on LB/LA Petri dish as described above. The amount of phage released into the supernatant was determined by infecting nonlysogenic MG1655 as described previously [8]. The amount of phage released per cell in culture was calculated as the PFU/CFU ratio. 

### 4.7. RecA-Mediated *in Vitro* Autoproteolysis

The standard buffer used in all assays contained 50 mM KCl, 15 mM Tris (pH 7.5), 2 mM MgCl_2_, 0.1 mM EDTA, 2 mM dithiothreitol. Concentrations of repressors from phage 434, λ, P22, and LexA in each reaction was 300 nm. λ repressor and anti-lambda repressor antibodies were a gift from Ann Hochschild (Harvard University Medical School, Cambridge MA, USA). LexA and anti-LexA antibodies were a gift from John Little, (University of Arizona, Tuscon, AZ, USA). Reaction mixtures were incubated 10 minutes at room temperature with different repressors, 0.5 mM γ-S-ATP, 2 mM DTT, and 3 μM oligo(dT_20_). After incubation, RecA protein was added to a final concentration of 8 µM, and the mixture was then incubated for 2 hours at 37 °C. Following incubation, Tris/Tricine loading dye (0.1 M Tris, pH 6.8, 1% sodium dodecyl sulfate (SDS), 20 mM 2-mercaptoethanol, 20% glycerol, 0.01% bromophenol blue) were added, and the samples were boiled for 5 min. The products were fractionated by electrophoresis on 15% SDS-polyacrylamide Tris-Tricine gels [33]. The repressor and its cleaved products were visualized by chemiluminescent detection of Western blots by a Storm Imager using an ECL-Plus kit (both obtained from GE Life Sciences, Piscataway, NJ, USA) with horseradish peroxidase secondary antibody.

### 4.8. Expression and Purification of Hex and sHex

A saturated, overnight culture of BL21(DE3)::pLysS with pMAL-c2X (New England Biolabs, Beverly, MA, USA), bearing the full Hex sequence, was diluted 1:50 in three liters of LB broth supplemented with 100 µg of ampicillin/mL and 20 µg of chloramphenicol/ml. The culture was grown for 2 hours at 37 °C. Cultures were induced to produce Hex by the addition of 0.5 mM IPTG. After additional growth for 4 hours at 37 °C, the cells were harvested by centrifugation at 10,000 × *g* for 10 minutes, and the cell pellet was resuspended in 30 mL of lysis buffer (100 mM Tris [pH 7.5], 200 mM NaCl, and 10 mM EDTA). All subsequent procedures were performed at 4 °C. The cells were lysed by using a French press, and the cell lysate was diluted to 100 mL with lysis buffer. The lysate was centrifuged at 10,500 × g for 20 minutes to remove cellular debris. The resulting supernatant was passed through a 2 mL Amylose resin column, washed three times with 5 bed volumes of resin buffer (100 mM Tris [pH 7.5], 500 mM NaCl), and eluted with resin buffer plus 10mM maltose in 500 µL fractions. The purification progress was monitored by analyzing fractions collected at each step by SDS-PAGE. MBP-Hex fractions were pooled and digested with factor Xa (Qiagen, Valencia, CA, USA) as specified by this manufacturer. Expected size of Hex peptide is ~17.5 kDa. The products were analyzed on 15% SDS-polyacrylamide Tris-Tricine gels. Unexpectedly, no full length Hex protein was observed. Instead, a ~6 kDa product was observed to accumulate. This product was cut out from the gel and its amino acid sequence was determined using MALDI-TOF MS analysis. Subsequently, DNA encoding this sHex (short Hex) was PCR amplified, cloned and expressed as described above. Purification of sHex was performed as described for the MBP-Hex fusion protein, except after breaking the cells and centrifuging the lysate at 10,500 × *g* for 20 minutes to remove cellular debris, sHex was precipitated from solution with addition of ammonium sulfate to the final concentration of 10%, and harvested by centrifugation at 10,000 × *g* for 10 minutes. The insoluble pellet was dissolved in final concentration of 4 M Guanidine-HCl (GuHCl) in 100 mM Tris [pH 7.5], 100 mM NaCl, and passed through 20 cm Sephadex G50 column equilibrated with the same buffer. The sHex containing fractions were pooled, concentrated, and dialyzed in buffers of successively lower concentrations of GuHCl in order to remove GuHCl. Purity of sHex was monitored by SDS-PAGE. 
